# Supplementary material for: Reduced Graphene Oxide-Zinc Sulfide Nanocomposite Decorated with Silver Nanoparticles for Wastewater Treatment by Adsorption, Photocatalysis and Antimicrobial Action
Source: Molecules. 2023 Jan 17;28(3):926. doi: 10.3390/molecules28030926 (PMC9920792; doi:10.3390/molecules28030926)
Supplement: Supplementary file 1 [file molecules-28-00926-s001.zip › molecules-2037960-supplementary.pdf]

Supplementary Material

Reduced graphene oxide-zinc sulfide nanocomposite decorated with silver nanoparticles for wastewater treatment by adsorption, photocatalysis and antimicrobial action

Hina Naeem[1], Hafiz Muhammad Tofil[2], Mohamed Soliman[3], Abdul Hai[4], Syeda Huma H. Zaidi[5], Nadeem Kizilbash[4], Daliyah Alruwaili[4], Muhammad Ajmal*[6], Muhammad Siddiq*[2]

[1]Department of Chemistry, Rawalpindi Women University, 6th Road, Satellite Town, Rawalpindi, Pakistan

[2]Department of Chemistry, Quaid-i-Azam University, Islamabad 45320, Pakistan

[3]Department of Microbiology, Faculty of Medicine, Northern Border University, Arar-91431, Saudi Arabia

[4]Department of Medical Laboratory Technology, Faculty of Applied Medical Sciences, Northern Border University, Arar-91431, Saudi Arabia

[5]Department of Chemistry, Faculty of Science, Northern Border University, Arar-91431, Saudi Arabia

[6]Department of Chemistry, Division of Science and Technology, University of Education, Lahore, Pakistan

*Correspondence:

Muhammad Ajmal, Email: m.ajmal65@yahoo.com, Phone Number: +92-3085513305, Department of Chemistry, Division of Science and Technology, University of Education, Lahore, Pakistan

Muhammad Siddiq, Email: m_sidiq12@yahoo.com, Phone Number: +92-51-90642147, Department of Chemistry, Quaid-i-Azam University, Islamabad 45320, Pakistan

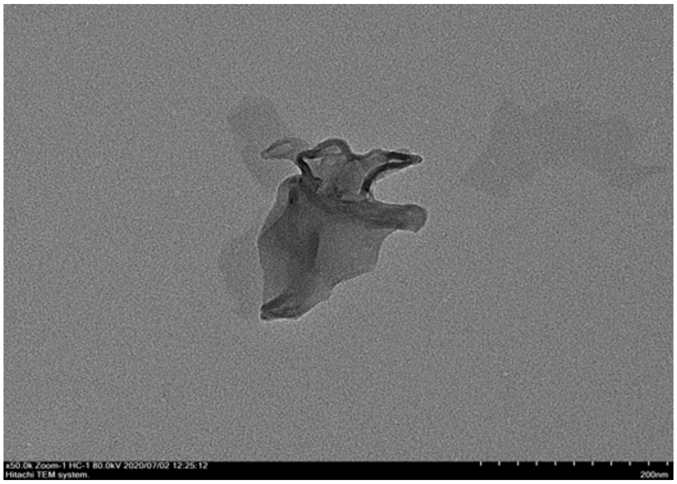

Figure S1. TEM image of rGO.

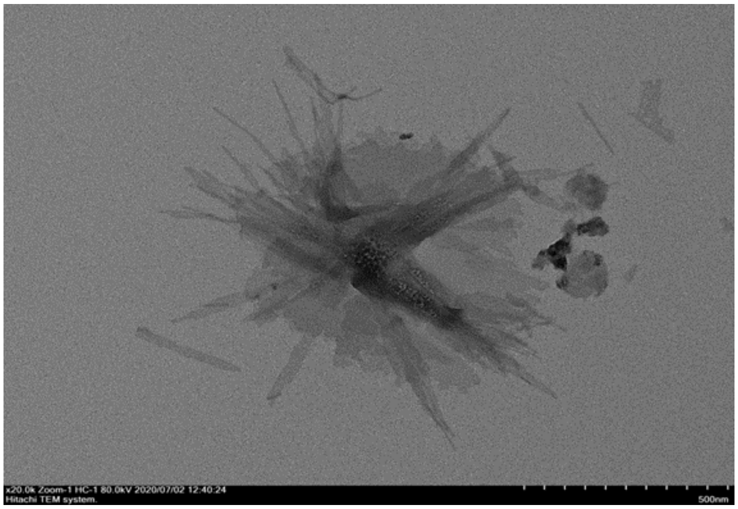

Figure S2. TEM image of rGO-ZnS-Ag nanocomposite.

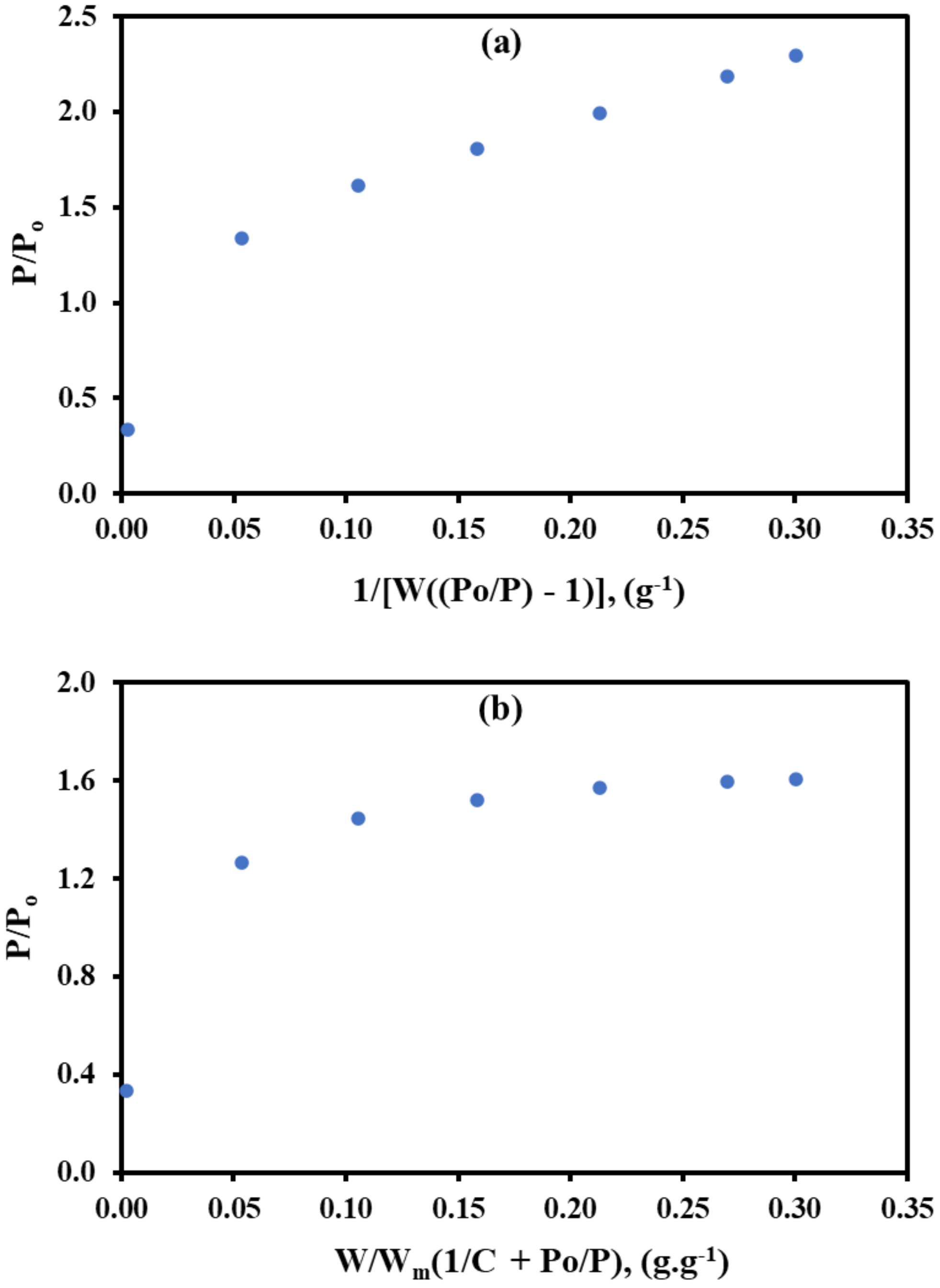

Figure S3. (a) BET and **(b)** Langmuir isotherm for Nitrogen gas adsorption on rGO-ZnS-Ag nanocomposite.

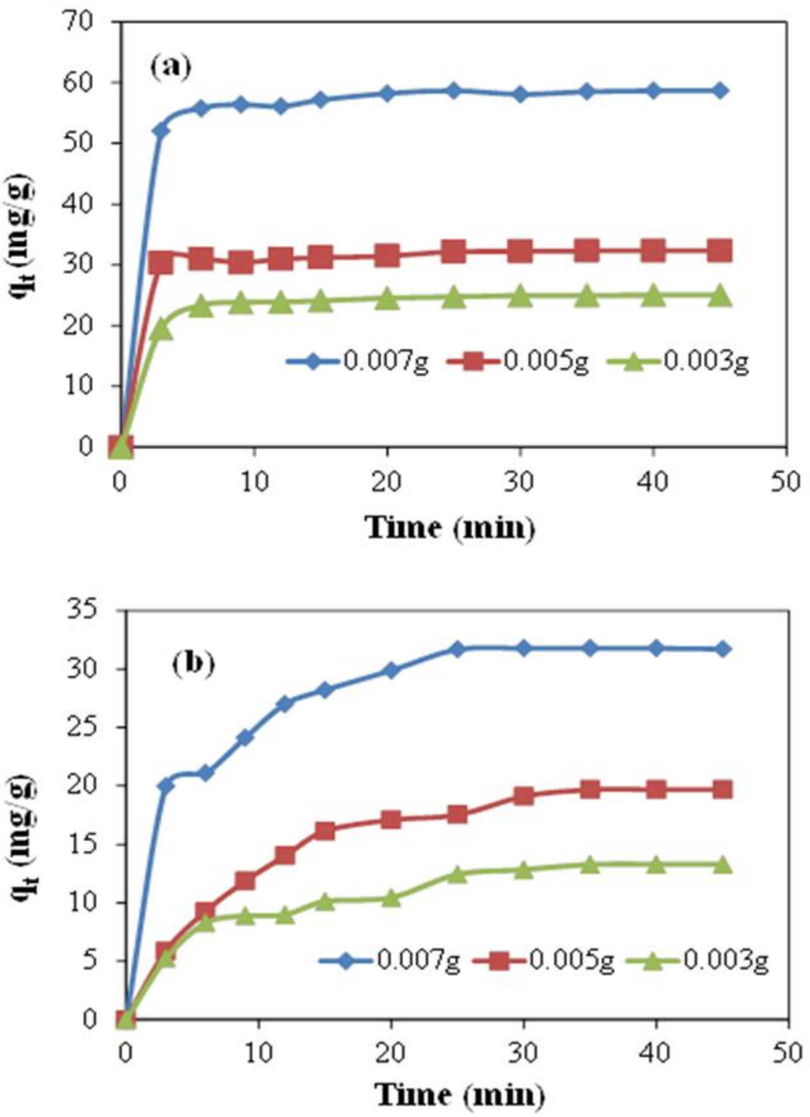

Figure S4: Effect of different amount of nanocomposite on the adsorbed amount of (a) MG and (b) EV.

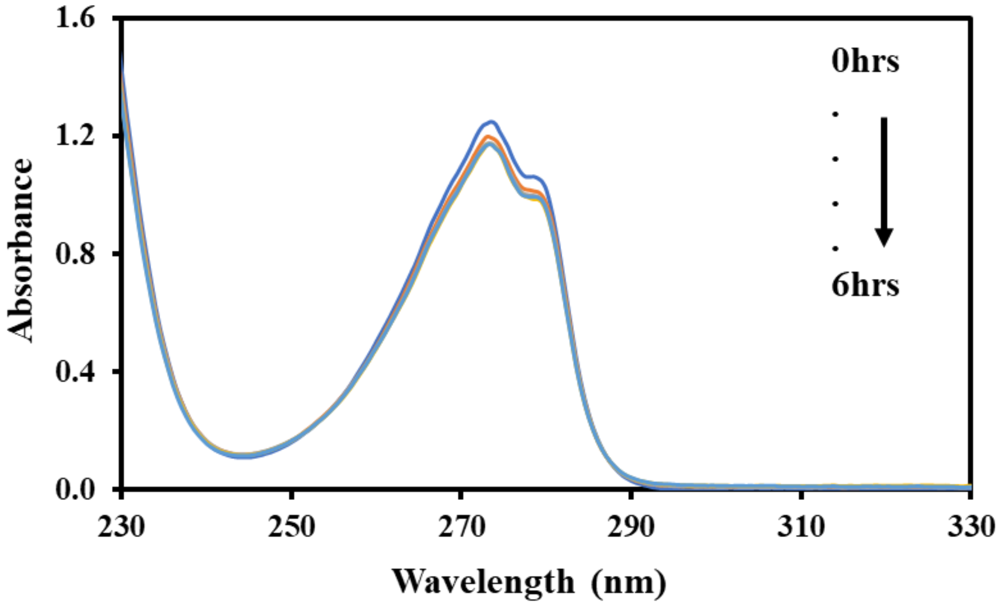

Figure S5: Photodegradation of resorcinol in the absence of catalyst.

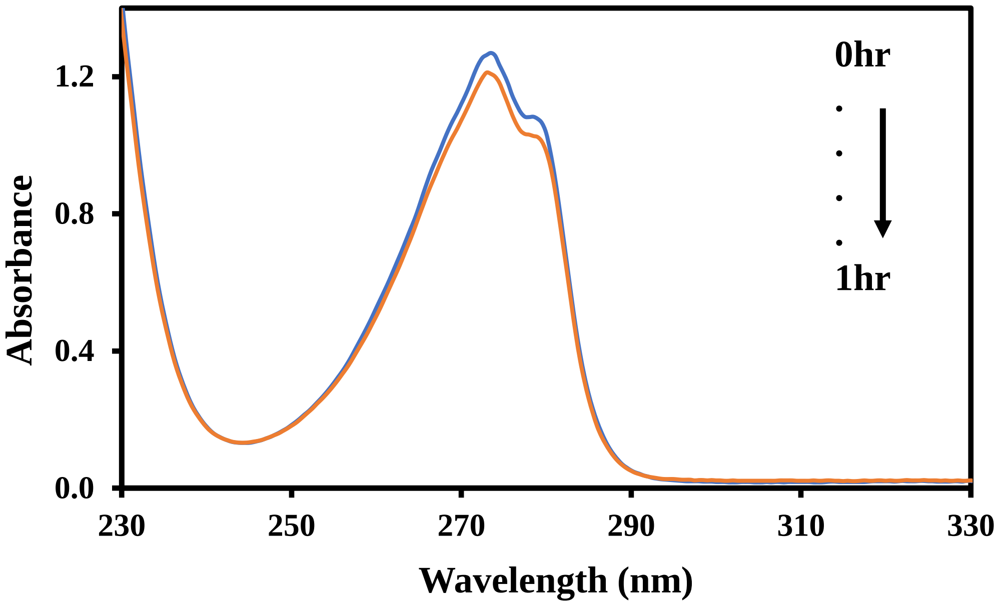

Figure S6: UV-Vis spectra of aqueous solution of resorcinol containing GO-Ag-ZnS nanocomposite as catalyst before exposing to light.

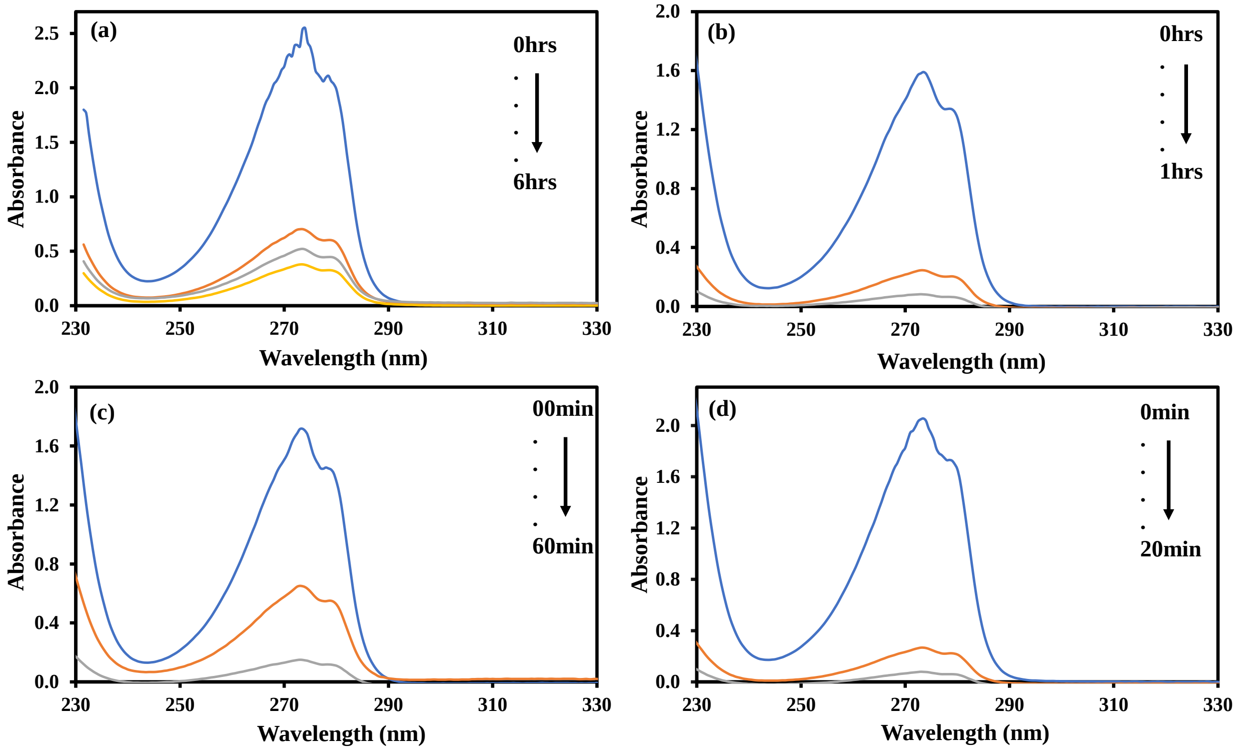

Figure S7: Rate of Resorcinol degradation at (a) 50 mg (b) 100 mg (c) 150 mg (d) 200 mg photocatalyst amount.
